# A nerve root decompression position identified by 3D CT scan: the modified reversed contralateral axial rotation position for patients with lumbar disc prolapse

**DOI:** 10.1186/s13018-025-05762-8

**Published:** 2025-04-17

**Authors:** Ahmed Raffet, Mark Laslett, Raymond Lee, Noha Khaled, Ghada Abdel Moneim Mohamed, Hossam Y. Sayed, Ahmed H. Omar, Maged M. Hawana, Mahmoud M. Ali, Salam M. Elhafez, Mohamed M. ElMeligie, Hossam Eddein Fawaz

**Affiliations:** 1 Department of Biomechanics, Faculty of Physical Therapy, Cairo University, Cairo, Egypt; 2 The Sports Clinic, 156 Bealey Avenue, Christchurch, 8014 New Zealand; 3 Department of Biomechanics, Faculty of Technology, Portsmouth University, Portsmouth, UK; 4 Department of Anatomy and Embryology, Faculty of Medicine, Cairo University, Cairo, Egypt; 5 Department of Neurosurgery, Faculty of Medicine, Cairo University, Cairo, Egypt; 6 Department of Radiology, Faculty of Medicine, Cairo University, Cairo, Egypt; 7 Department of Physical Therapy for Neurology and its Surgery, Faculty of Physical Therapy, Cairo University, Cairo, Egypt; 8 Department of Basic Sciences for Physical Therapy, Faculty of Physical Therapy, Ahram Canadian University, Giza, Egypt; 9 Present Address: Department of Basic Sciences for Physical Therapy, Faculty of Physical Therapy, Al Hayah University, Cairo, Egypt

**Keywords:** Lumbar intervertebral foramen, Nerve root decompression position, Modified reversed contralateral axial rotation position, Lumbar disc prolapse, Radiculopathy

## Abstract

**Background:**

Nerve root compression in the lumbar intervertebral foramen (LIVF) is a consistent feature of radicular syndrome. There is debate about movements and positions that may reduce compression for possible use in conservative treatment.

**Purpose:**

To investigate real-time effects of specific 3 dimensional positioning of the trunk on the cross sectional area (CSA) of the LIVF in patients with lumbar disc prolapse and radiculopathy using 3D-CT scan imaging.

**Methods:**

Ninety males aged between 20 and 40 years with unilateral lumbar disc prolapse and radiculopathy were separated into three equal groups based on the level of disc prolapse. Group (A): L3/L4, group (B): L4/L5, and group (C): L5/S1. All underwent three separate imaging sessions; first in the supine position to establish baseline data (Baseline-Image 1), followed by a modified reversed contralateral axial rotation position (Image 2), and finally the same position as Image 2 but after 48 h of using the position as a therapeutic intervention (Image 3). The CSA of LIVF at L3/L4, L4/L5, and L5/S1 levels and the angles of straight leg raising (SLR) test were measured following each imaging session.

**Results:**

Two-way mixed MANOVA analysis revealed that the mean values of the CSA of LIVF and the angle of SLR test were significantly increased in Image 2 compared with Baseline-Image 1 across all tested groups (*P* = 0.001). Moreover, the measured outcome variables were significantly increased in Image 3 compared with Image 2 and Baseline-Image 1 across all tested groups (*P* = 0.001).

**Conclusion:**

The modified reversed contralateral axial rotation position of the trunk had a real-time decompression effect on the impinged nerve roots in patients with unilateral lumbar disc prolapse and radiculopathy.

## Introduction

The lumbar intervertebral foramen (LIVF) is the doorway where lumbar nerve roots and mixed spinal nerves can easily get compressed due to various pathological disorders [1]. Radiculopathy is a neuromechanical condition characterized by nerve root compression and foraminal constriction [2]. As a mechanical lesion, disc herniation is the major pathophysiology that tends to impact the nerve root [3].

The dimensions of LIVF constantly change throughout daily activities [4]. Therefore, symptoms of nerve root compression can be exacerbated or attenuated by the posture of the lumbar spine. One of the conservative nerve root decompression approaches is a manual orthopaedic physiotherapy technique known as positional decompression [5]. This position may provide clinically significant pressure relief for the nerve roots. In 1993, Cyriax [6] introduced the “reverse rotation strain” position as a manipulation position for the first time, which involves lumbar axial rotation in the transverse plane. In 1996, Winkel et al. [7] utilized this position in “axial separation in flexion” as a mobilization technique applied with an emphasis on axial separation, rotation, or a combination of the two. In 2003, Ombregt et al. [8] used this position again as a “reverse stretch” manipulation technique. In 2018, Sabbahi and Ovak-Bittar [2] tested the H-reflex amplitude to assess the nerve root compression/decompression effects using this position while treating a forty-year-old woman suffering from lower back pain and radiculopathy. However, all previous researchers did not explore the impact of compression/decompression effects on the impinged nerve roots through specific examinations of LIVF dimensions under computed tomography (CT) scan.

Earlier research on the anatomical characteristics of the LIVF was conducted using fresh cadaver specimens in a laboratory setting but was constrained by the absence of muscle tone [9, 10]. In addition, when creating computational models, the biomechanical approaches for studying the lumbar spine did not consider the primary restraints of spinal motion (tendons and muscles) [11, 12]. In-vivo, magnetic resonance imaging (MRI) may not always offer comprehensive information and could potentially yield inaccurate results [13]. Advancements in computer technology and digitization have allowed for the examination of the three-dimensional (3D) morphological features of intricate anatomical structures, resulting in enhancements in evaluating spinal biomechanics [14].

In the current study, a mechanical modification of the reverse rotation strain position was performed, producing the modified reversed contralateral axial rotation (MRCAR) position. To the best of our knowledge, there have been no prior studies investigating the in-vivo anatomical cross sectional area (CSA) of the LIVF from the MRCAR position, which involves a combination of lumbar axial rotation, side bending, and flexion in the transverse, frontal, and sagittal planes, respectively. The aim of this research was to explore the real-time and short-term influence of the MRCAR trunk posture on the CSA of the LIVF and straight leg raising (SLR) range in patients with unilateral lumbar disc prolapse and radiculopathy, using 3D-CT scan imaging.

## Materials and methods

### Participants

A convenient sample of ninety male patients diagnosed with unilateral lumbar disc prolapse and radiculopathy were assigned to one of three equal groups based on the disc prolapse level; group (A): L3/L4, group (B): L4/L5, and group (C): L5/S1. Patients aged 20 to 40 were recruited to minimise additional age-related impacts on the spine and disc properties after age 40 [15]. All had a “second-grade” paracenteral or foraminal disc bulge (2–3 mm) according to Fardon and Milette [16], detected by T2 MRI axial views. No evidence of disc extrusion was present, where the disc material remained confined between the adjacent endplates of the parent disc. The pedicle to vertebral body height ratio was equal to or less than 0.5 mm [17]. The patients were experiencing lower back pain and unilateral radiculopathy for at least three months, and all had a positive SLR test. The diagnosis was verified through physical and neurological examinations including motor and sensory evaluations as well as reflex testing. Only male patients were involved to prevent the impact of hormonal fluctuations, specifically relaxin, which rises during the menstrual cycle in females. Relaxin can affect musculoskeletal flexibility and spinal mobility, causing laxity of neural foraminal ligaments of the lumbar spine, which potentially changing movement patterns of the lumbar spine and, consequently, confounding biomechanical data when evaluating an anatomical opening as small as the LIVF on a 3D-CT scan [18]. Patients with bilateral and multilevel prolapses, sequestrated, migrated, calcified, and herniated discs, posterior apophyseal ring separation, Modic changes, piriformis syndrome, sacroiliac joint dysfunction, and acute onset of pain were excluded.

Patients underwent 3D-CT scan imaging at a radiology center specializing in the spine. Those meeting the inclusion criteria agreed to sign an informed consent form. This research was authorized by the Research Ethical Committee of the Faculty of Physical Therapy, Cairo University (Approval/code/NO: P.T.REC/012/004981) and the ClinicalTrial.gov public website (Approval/code/NO: NCT06359470).

### Instrumentation

Lumbar 3D-CT scan was performed using Philips Brilliance 64 Multi Slice CT Scanner. The DoseWise™ technology targets the lowest dose with very high image resolution. All data was obtained using an integrated positron emission tomography and computed tomography (PET/CT) system. This specialized system combines a PET scanner with a multi-section helical CT scanner, allowing for the capture of CT and PET images simultaneously. The CT technologist was well-trained and qualified to ensure that radiation doses are kept as low as reasonably achievable (ALARA) in every scan, in order to obtain high-quality images with minimized radiation exposure.

### Imaging

The 3D-CT scan images were acquired on three occasions:

#### Baseline-Image 1

**Fig. 1:**
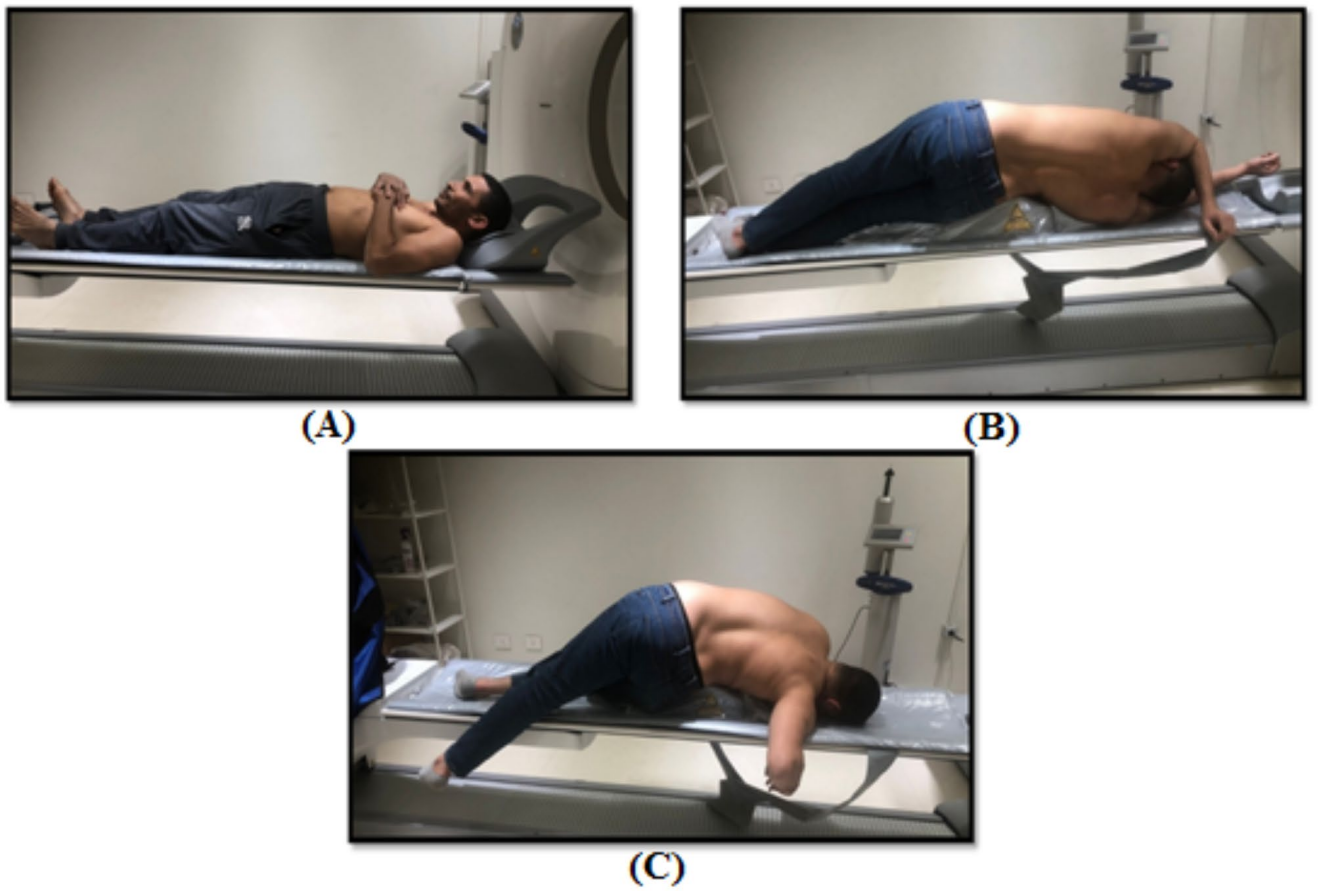
Body positions during 3D-CT scan imaging of asymptomatic individuals: (**A**) Conventional neutral supine position. (**B**) Lumbar side bending toward the pain-free side. (**C**) Modified reversed contralateral axial rotation (MRCAR) position; lumbar side bending and rotation toward the pain-free side

The first image was captured in the conventional neutral supine position, providing baseline data (Fig. 1-A).

#### Image 2

**Fig. 2:**
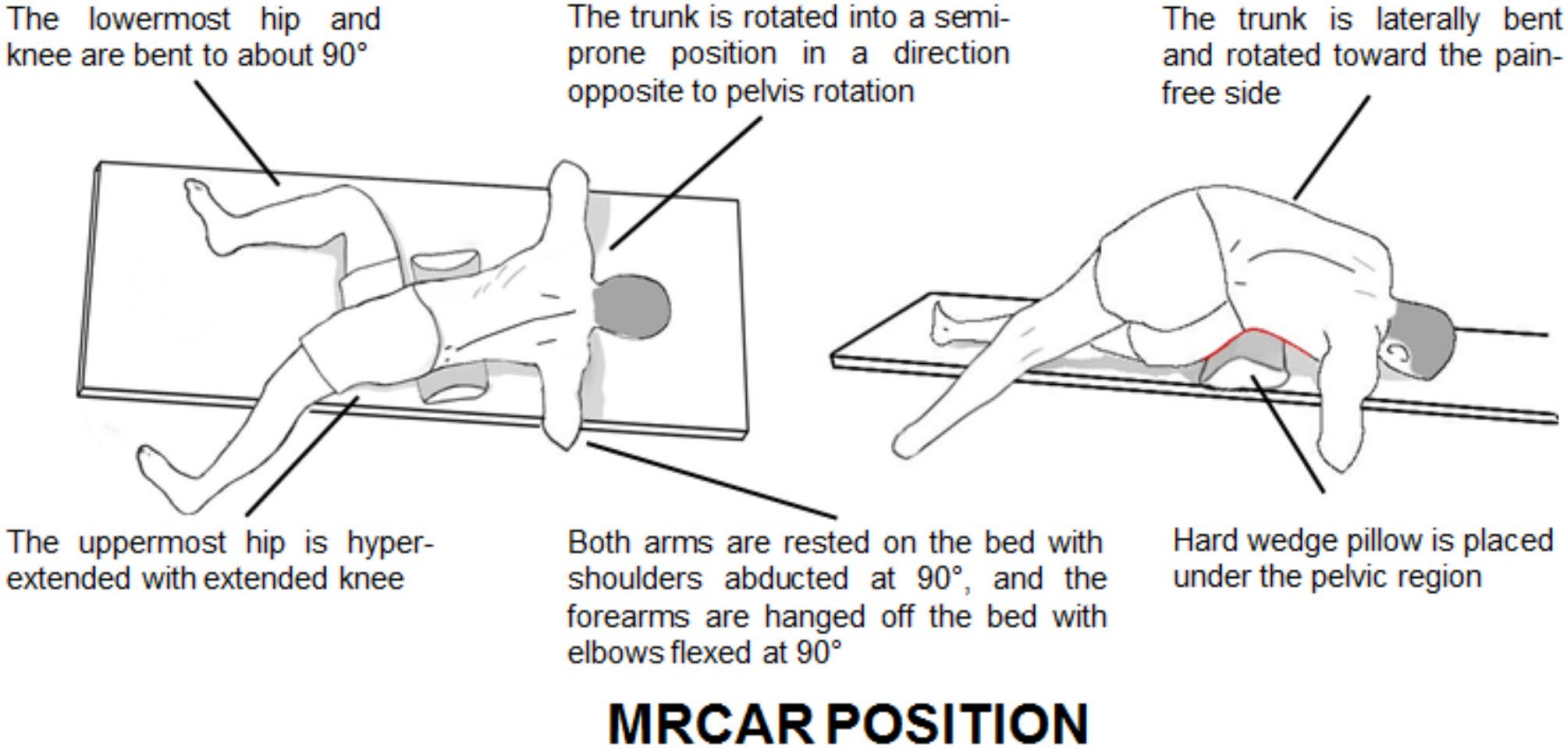
Modified reversed contralateral axial rotation (MRCAR) position; sagittal view on right and transverse view on left

Immediately following acquisition of Image 1, the patient assumed a side lying position on the pain-free side. This position was standardized by placing a hard wedge pillow measuring 30 cm in height and 75 cm in length beneath the pelvic region, keeping the upper edge of the wedge pillow base just above the level of the iliac crest for all patients (Fig. 1-B). The patient was then positioned in the MRCAR posture by rotating the trunk away from the painful side opposite to pelvis rotation, while ensuring the lower shoulder and upper pelvis remained aligned. Both arms were resting on the bed with shoulders abducted at 90°, while the forearms were hanging off the bed with elbows flexed at 90°. The hip and knee of the uppermost limb were fully extended, while the lowermost were flexed to approximately 90° (Figs. 1-C and 2). The second image was acquired in this position.

#### Image 3

The third image was in the same MRCAR posture and was acquired 48 h later.

### Images interpretation

**Fig. 3:**
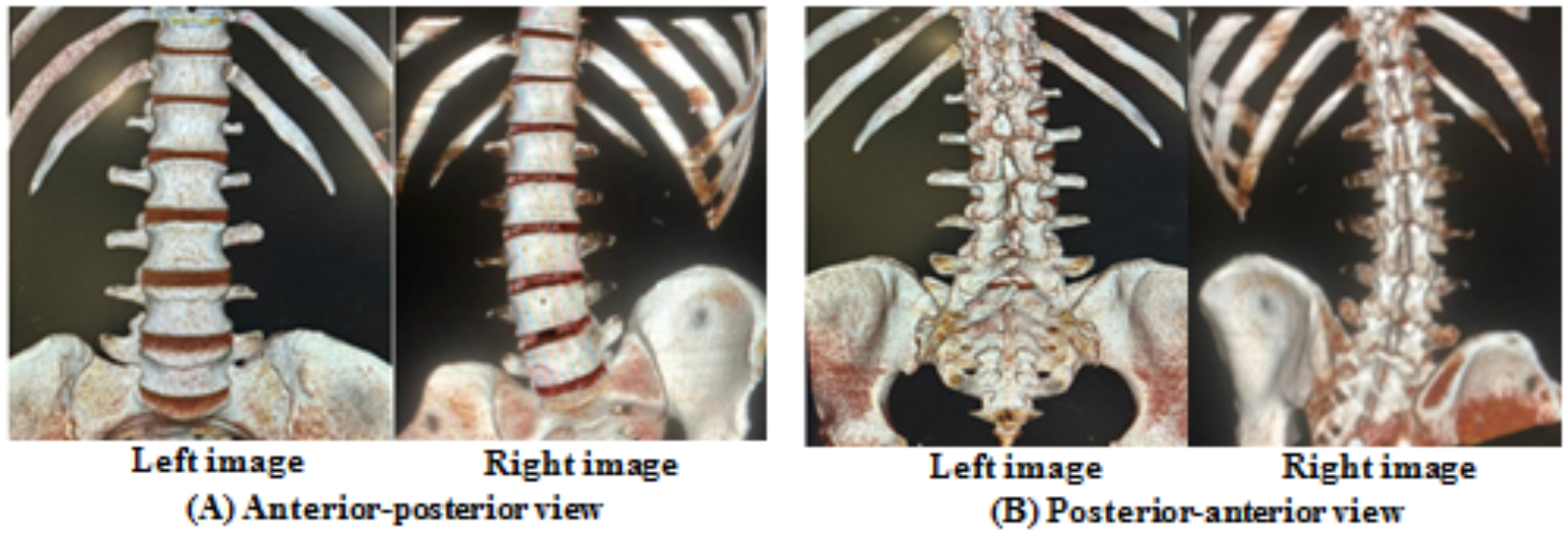
Lumbar 3D-CT scan images; right images from the modified reversed contralateral axial rotation (MRCAR) position and left images from the supine position

**Fig. 4:**
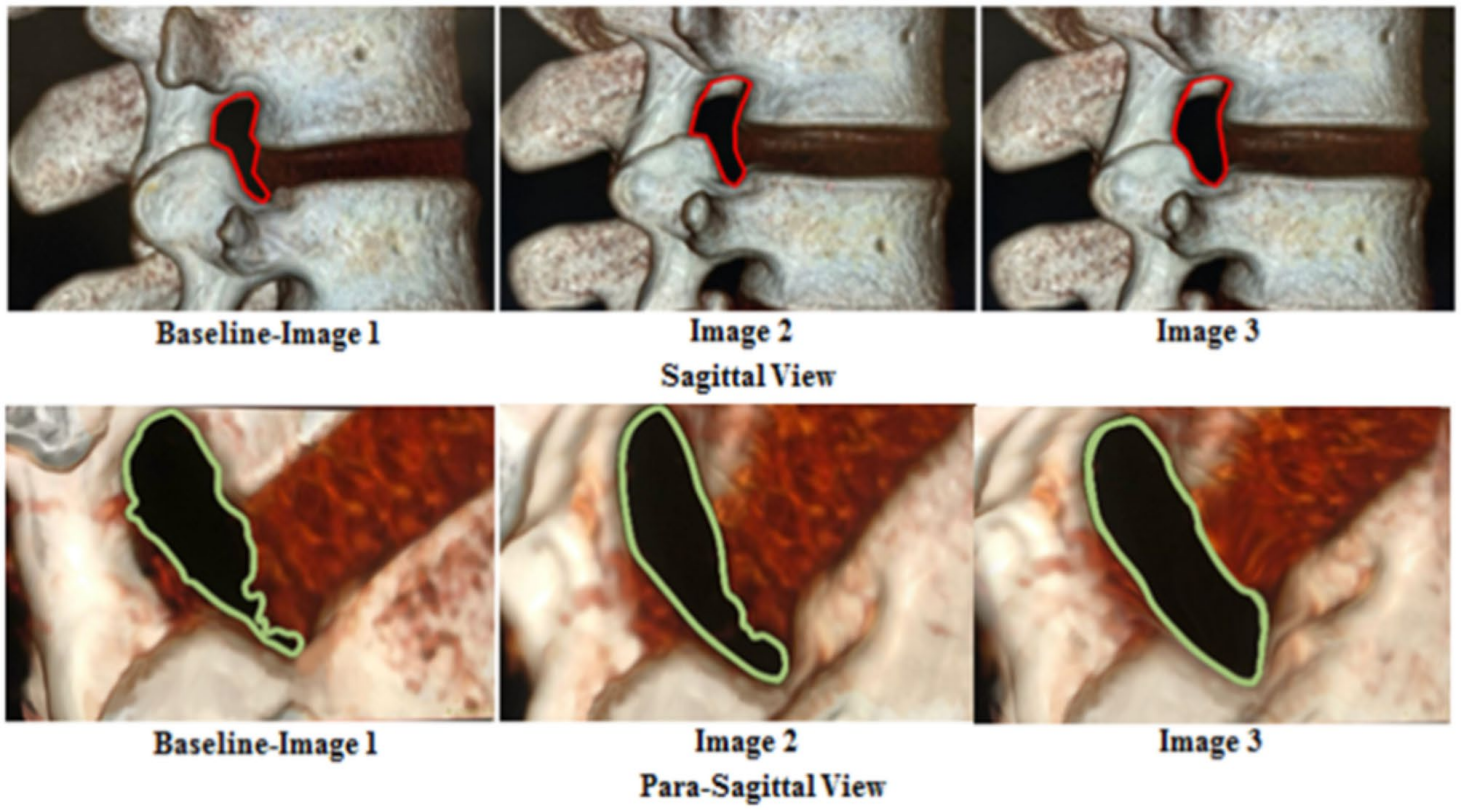
3D-CT scan images of the CSA of the LIVF at L5/S1 level

An experienced radiologist analyzed all the 3D-CT images. The CSA of the LIVF (cm²) was measured from the true sagittal plane at the L3/L4, L4/L5, and L5/S1 levels using the boundaries of the neighboring superior and inferior pedicles, the posterosuperior part of the lower vertebral body, the back part of the intervertebral disc, the posteroinferior part of the upper vertebral body, and the front part of ligamentum flavum. The lumbar 3D-CT scan images and the CSA of the LIVF are displayed in (Figures 3 and 4), respectively.

### Therapeutic intervention

All patients were educated and instructed to adopt the MRCAR position 4 times daily, each lasting 20 min, for a period of 48 h between Image 2 and Image 3 acquisitions. As the hip and knee of the uppermost limb were fully extended, the patients were educated to exert a gentle post-end-range push in backward and downward directions as much as possible to ensure the optimal and maximal positioning (i.e., self-augmented maneuver).

### The straight leg raising (SLR) test

Upon finishing the images captured during the previous 3D-CT scan occasions, the patient’s angle of SLR test (degree) was assessed to detect nerve root irritation and potential entrapment causing reduced nerve excursion. It exerts mechanical tension on the L4, L5, S1, S2, and S3 nerve roots, primarily focusing on the L5 and S1 roots [19]. It was assessed as an initial baseline response following Image 1, then measured after Image 2, and finally judged the therapeutic intervention’s effectiveness following Image 3. The SLR test of the affected lower limb was performed from the supine position. The examiner placed one hand on the patient’s knee, keeping it extended, and the other hand holding the heel. The patient’s leg was then slowly lifted until the pain was felt anywhere in the affected limb. Once the patient was reported feeling pain, another examiner measured the angle between the bed and the affected limb using a universal goniometer.

### Data analysis

The measured outcome variables were the CSA of LIVF (cm²) and the angle of SLR test (degree). Initially, data underwent tests for normality such as Shapiro-Wilk and Kolmogorov-Smirnov, as well as checks for skewness, kurtosis, box plots, and frequency distribution curves. These tests indicated that the data followed a normal distribution, leading to the use of parametric analysis. The Statistical Package for Social Science (SPSS) version 25 for windows was utilized for conducting all statistical computations. The significance level was established at 0.05.

### Statistical analysis

One-way between-subject Analysis of Variance (ANOVA) was carried out to reveal any significant variations in the baseline eligibility criteria of the sample. Mixed-design Multivariate Analysis of Variance (MANOVA) was employed to compare the results of the two measured variables of interest among the three 3D-CT scan occasions (Baseline-Image 1, Image 2, and Image 3) for all tested groups. Multiple pairwise comparison post-hoc tests were conducted afterwards to identify the source of significance for each measured variable. The effect sizes were likewise assessed utilizing Cohen’s d.

## Results

One-way between-subject ANOVA reveal that the mean values of age, body mass, height, and BMI were homogeneous among the three tested groups (*p* > 0.05) as shown in Table 1. Descriptive statistics illustrated the mean values of the measured outcome variables are presented in Tables 2 and 3. Data are expressed by mean ± standard deviation (SD).

**Table 1:** Sample descriptive statistics

	Group A (L3/L4)	Group B (L4/L5)	Group C (L5/S1)	F-value	*P*-value
Age (years)	35.91 ± 3.74	35.44 ± 4.16	35.23 ± 4.92	0.198	0.820
Body mass (kg)	87.45 ± 12.71	88.35 ± 12.24	88.9 ± 12.01	0.110	0.896
Height (cm)	177.80 ± 8.06	180.43 ± 6.22	179.03 ± 6.08	1.110	0.334
BMI (kg/m²)	27.53 ± 2.31	27.07 ± 2.84	27.66 ± 2.62	0.426	0.654

Data presented as mean ± SD

**Table 2:** Descriptive statistics of the CSA of LIVF (cm^2^)

	CSA of LIVF (cm2)		
	Baseline (Image 1)	Image 2	Image 3
Group A	0.255 ± 0.064	0.369 ± 0.085	0.691 ± 0.143
Group B	0.171 ± 0.063	0.296 ± 0.161	0.566 ± 0.262
Group C	0.134 ± 0.071	0.202 ± 0.094	0.441 ± 0.171

Data presented as mean ± SD

**Table 3:** Descriptive statistics of the angle of SLR test (degree)

	Angle of SLR test (degree)		
	Baseline (Image 1)	Image 2	Image 3
Group A	41.06 ± 10.49	44.80 ± 11.36	65.83 ± 10.85
Group B	35.20 ± 5.80	41.53 ± 6.63	69.63 ± 8.84
Group C	29.26 ± 6.92	35.93 ± 6.65	65.23 ± 6.94

Data presented as mean ± SD

**Fig. 5:**
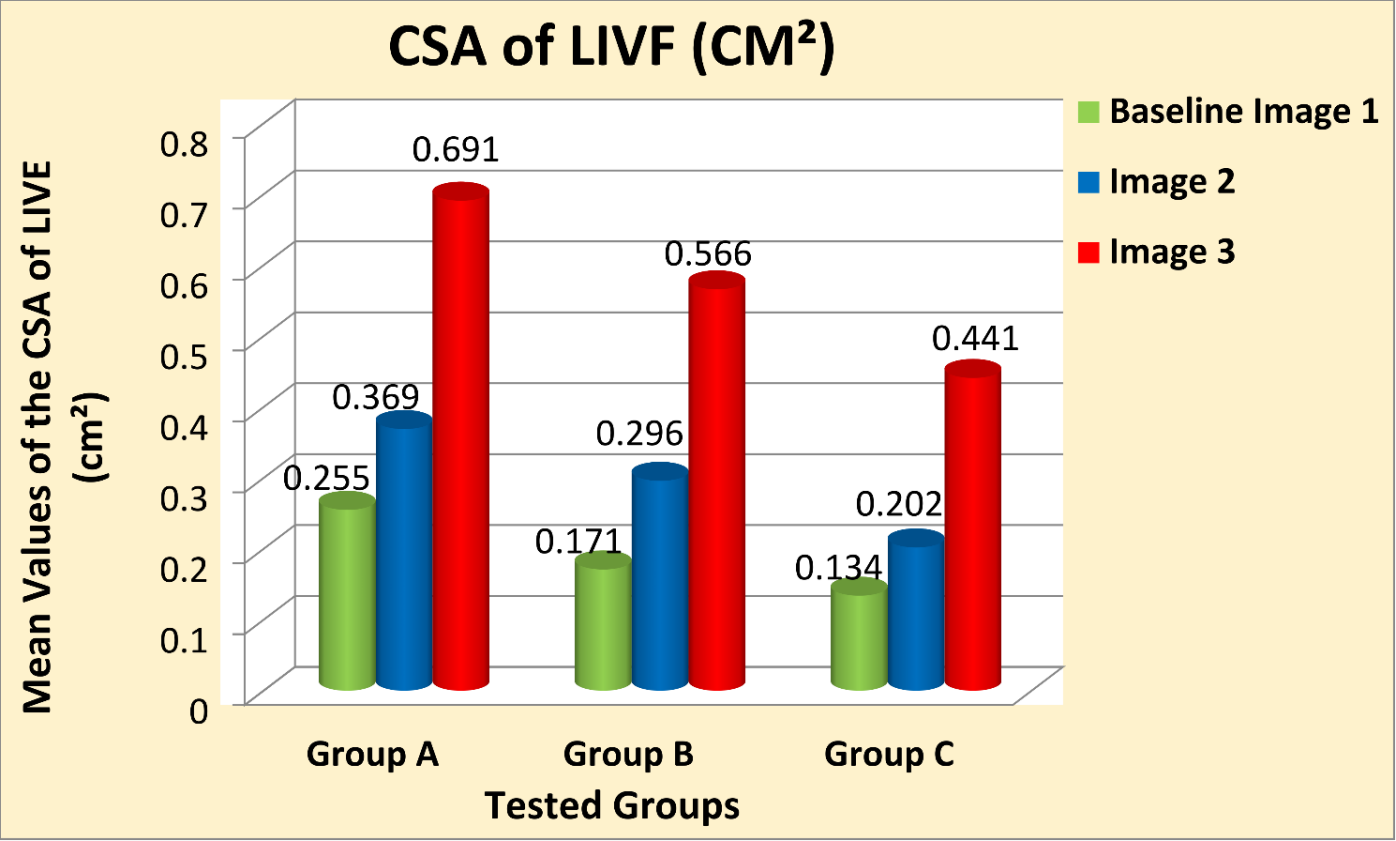
Mean values of the CSA of the LIVF (cm^2^) at the three 3D-CT scan images different tested groups

**Fig. 6:**
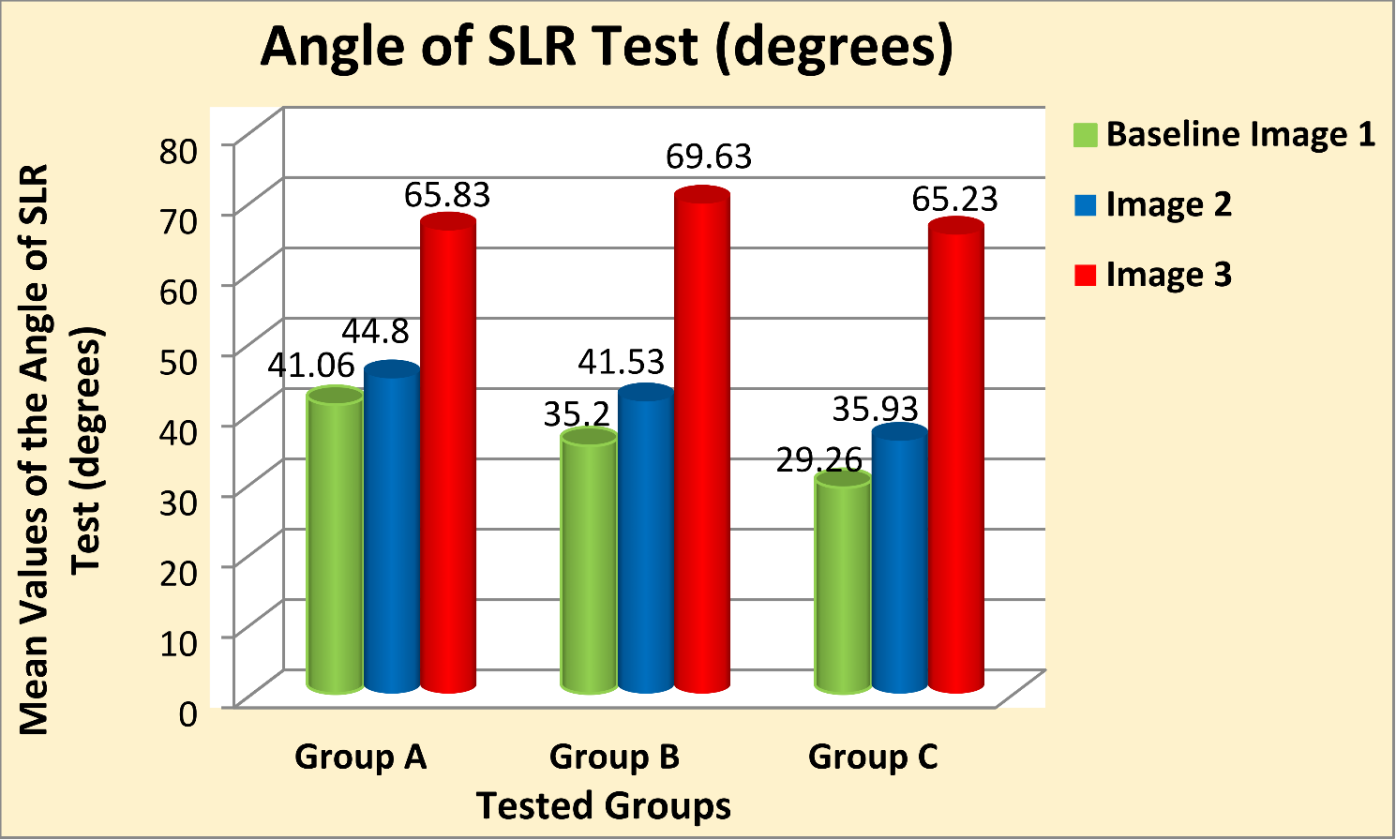
Mean values of the angle of SLR test (degrees) after the three 3D-CT scan images at different tested groups

Mixed-design MANOVA analysis showed significant effects of the three 3D-CT scan images on the measured variables of interest (*P* = 0.001). Multiple pairwise comparison post-hoc tests revealed that the mean values of the CSA of LIVF (cm²) and the angle of SLR test (degree) were significantly increased in (Image 2) compared with (Baseline-Image 1), with a large effect size across all tested groups (*p* = 0.001, Cohen’s d > 0.8) except for the small effect size of the SLR test angle in group A (*p* = 0.001, Cohen’s d = 0.34). Moreover, the measured variables showed a significant increase in (Image 3) in comparison to (Image 2) and (Baseline-Image 1), with a large effect size across all tested groups (*p* = 0.001, Cohen’s d > 0.8) (Table 4) (Figs. 5 and 6).

**Table 4:** Multiple pairwise comparison post-hoc tests and Cohen’s d effect size of the CSA of LIVF (cm^2^) and the angle of SLR test (degree)

CSA of LIVF (cm^2^)												
3D-CT Scan Images	Group A				Group B				Group C			
	**p-value**	**Cohen’ d**	**95% CI**		**p-value**	**Cohen’ d**	**95% CI**		**p-value**	**Cohen’ d**	**95% CI**	
			**Low**	**High**			**Low**	**High**			**Low**	**High**
Image 1 vs. Image 2	0.001*	**1.515**	**0.07**	**0.15**	0.001*	**1.022**	**0.08**	**0.16**	0.001*	**0.816**	**0.02**	**0.11**
Image 1 vs. Image 3	0.001*	**3.935**	**0.35**	**0.51**	0.001*	**2.073**	**0.35**	**0.51**	0.001*	**2.344**	**0.22**	**0.38**
Image 2 vs. Image 3	0.001*	**2.737**	**0.27**	**0.36**	0.001*	**1.241**	**0.22**	**0.31**	0.001*	**1.732**	**0.19**	**0.28**
**Angle of SLR test (degree)**												
Image 1 vs. Image 2	0.001*	**0.342**	**2.58**	**4.88**	0.001*	**1.016**	**5.18**	**7.48**	0.001*	**0.982**	**5.51**	**7.81**
Image 1 vs. Image 3	0.001*	**2.32**	**21.2**	**28.2**	0.001*	**4.605**	**30.9**	**37.9**	0.001*	**5.190**	**32.4**	**39.4**
Image 2 vs. Image 3	0.001*	**1.893**	**17.5**	**24.4**	0.001*	**3.596**	**24.6**	**31.5**	0.001*	**4.311**	**25.8**	**32.7**

*Statistically significant when P value is less than 0.05

## Discussion

The MRCAR position significantly increases the CSA of the LIVF immediately. When used as a therapeutic intervention for 48 h, the MRCAR position resulted in further improvements in the CSA of the LIVF and the nerve root mobility as measured by the SLR test. These increases are clinically relevant and have practical importance owing to the large effect size noted in the research findings of the measured variables across all groups at the three 3D-CT scan occasions.

Lumbar radiculopathy is predominantly a consequence of disc-induced nerve root impairment [20]. Compression and impediment to normal movement of the nerve root occurs when the LIVF space is crowded by the displaced disc [21]. Although LIVF stenosis is clinically important, there is limited in-vivo data available on how LIVF geometry changes in specific trunk positions. As far as we know, this is the first in-vivo study investigating the effect of the MRCAR position of the trunk on the CSA of the LIVF in patients with unilateral lumbar disc prolapse and radiculopathy using 3D-CT scan technology.

Measuring the LIVF dimensions could also be used to track progression of a patient’s condition over time. The imaging technique described here provides objective data for assessing treatment efficacy through changes in the LIVF CSA [15]. The findings of the current study reveal a statistically significant immediate and sustained increase in the LIVF CSA in the MRCAR position when compared with the supine position across all tested groups. It establishes the potential merit of positional decompression as a treatment position opening the LIVF and relieving nerve root compression.

**Fig. 7:**
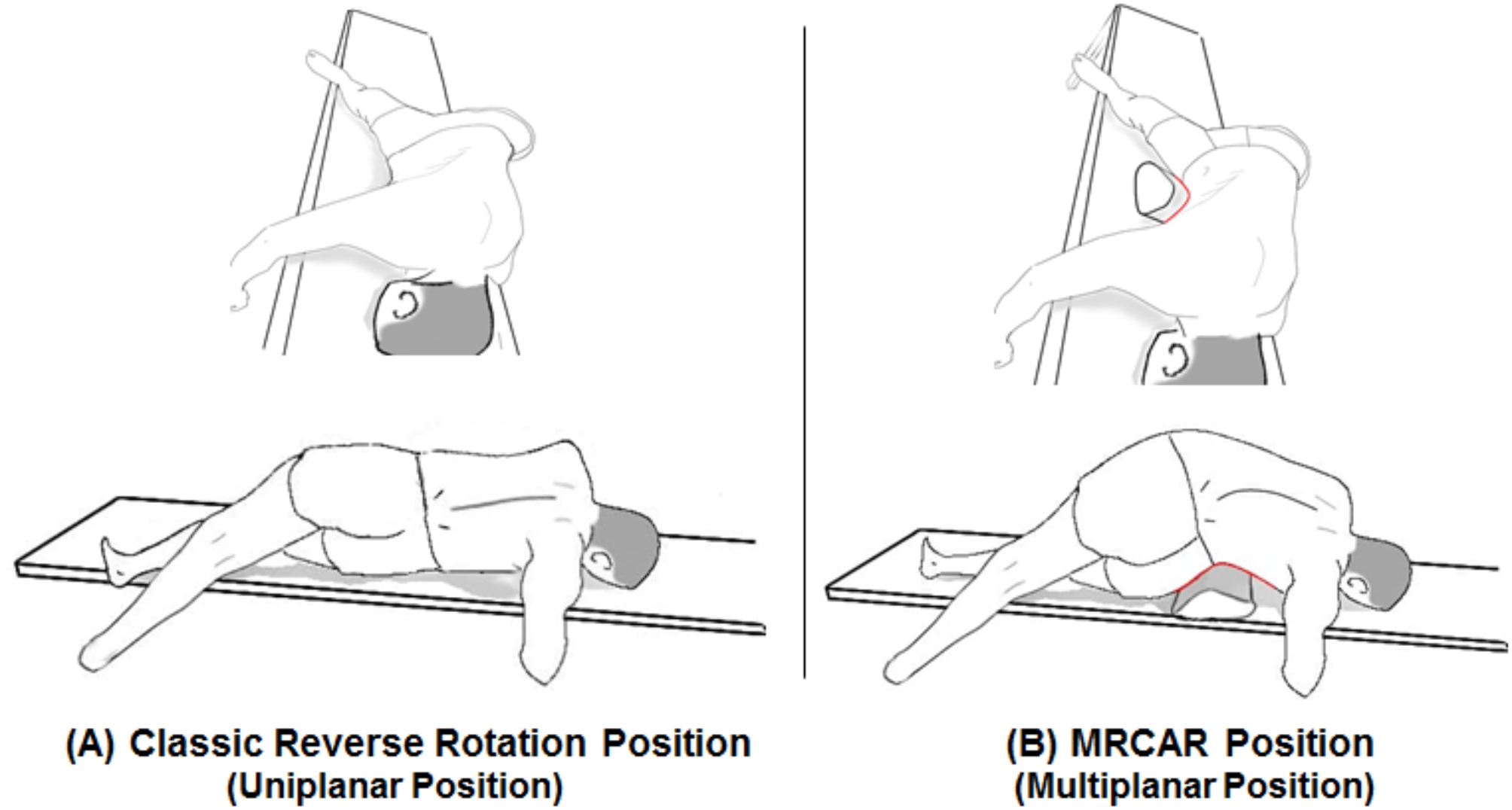
(**A**) Classic reverse rotation strain position involves lumbar axial rotation in the transverse plane. (**B**) Modified reversed contralateral axial rotation (MRCAR) position combines lumbar axial rotation, side bending, and flexion in the transverse, frontal, and sagittal planes, respectively

**Fig. 8:**
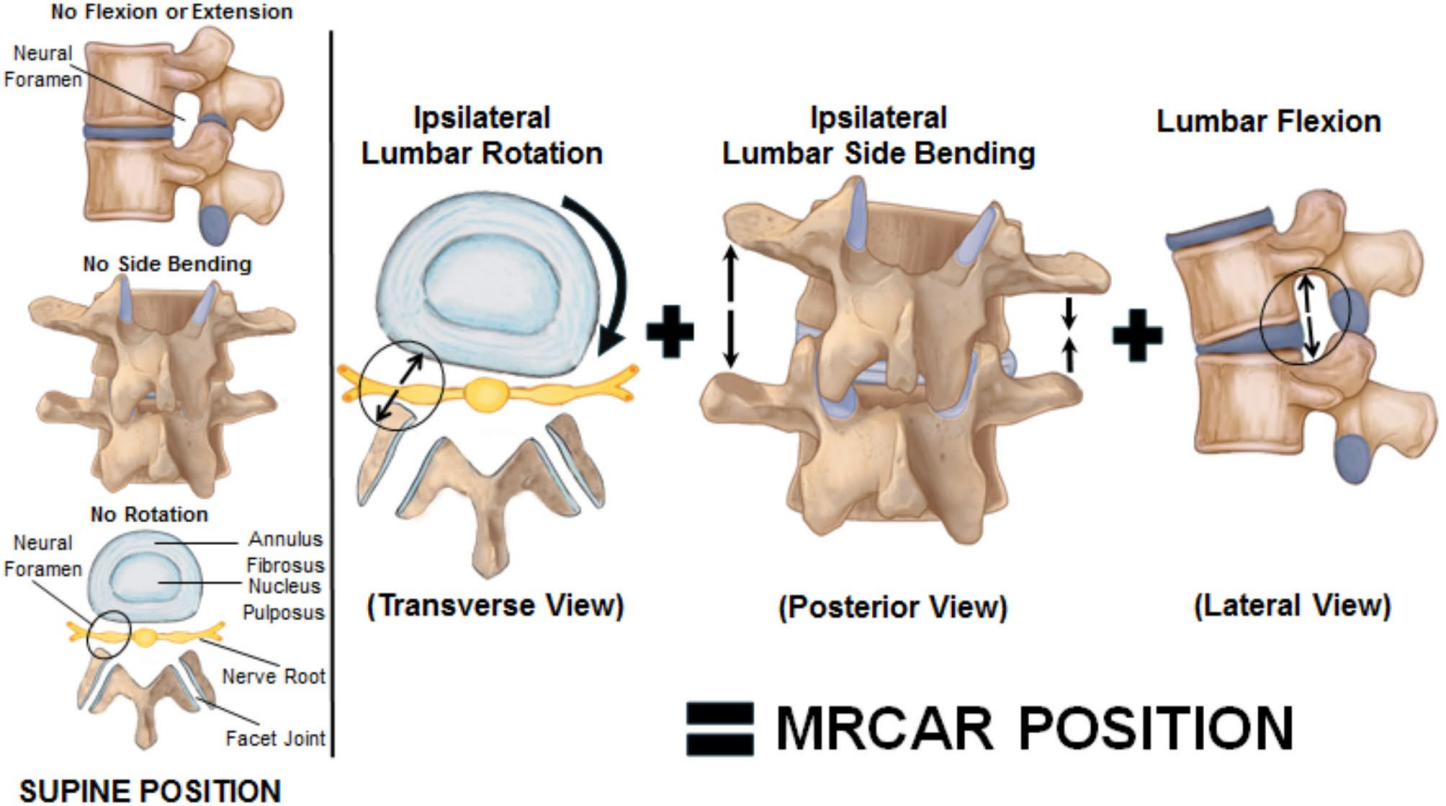
Schematic model illustrates that the modified reversed contralateral axial rotation (MRCAR) position effectively opens the foramen by incorporating lumbar axial rotation, side bending, and flexion in the transverse, frontal, and sagittal planes, simultaneously

Previous research has examined the morphological alterations in the LIVF during lumbar flexion, side bending, and axial rotation [4, 22–25]. It has been established that flexion led to a significant increase in the width, height, and area of the LIVF, while side bending resulted in a significant increase in the width, height, and area of the LIVF on the opposite side of bending, and axial rotation led to a significant increase in the height and area of the LIVF on the opposite side of rotation. Moreover, the LIVF was opened by 11.3% with lumbar flexion, 8.0% with side bending, and 6.5% with axial rotation [4]. In the current study, combination of these uniplanar postures was studied with the MRCAR position (Fig. 7), and appeared to be a potent foraminal opening position (Fig. 8) resulting in sustained nerve root decompression and improved nerve root mobility. It is therefore of interest as a manual therapy technique for managing lumbar disc prolapse and radiculopathy.

The findings of the present study are also consistent with earlier researches. Creighton stated that lying on one side over a six-inch diameter towel roll improved the anteroposterior and vertical opening of the LIVF at the opposite side of lumbar bending [26]. Singh et al. reported that some positions, such as lumbar extension, may reduce the LIVF dimensions and cause potential spinal nerve root injury, while others, such as flexion or side bending, may increase LIVF dimensions and reduce the nerve root compression [22]. Sabbahi and Ovak-Bittar used the reverse rotation strain treatment position for a 40-year-old female who had L4/L5 disc herniation and left radiculopathy and found that the increased H-reflex amplitude after treatment shows the electrophysiological proof of neural decompression linked to axonal recovery, associating with an increase in the SLR angle from 30° to 90° [2].

**Fig. 9:**
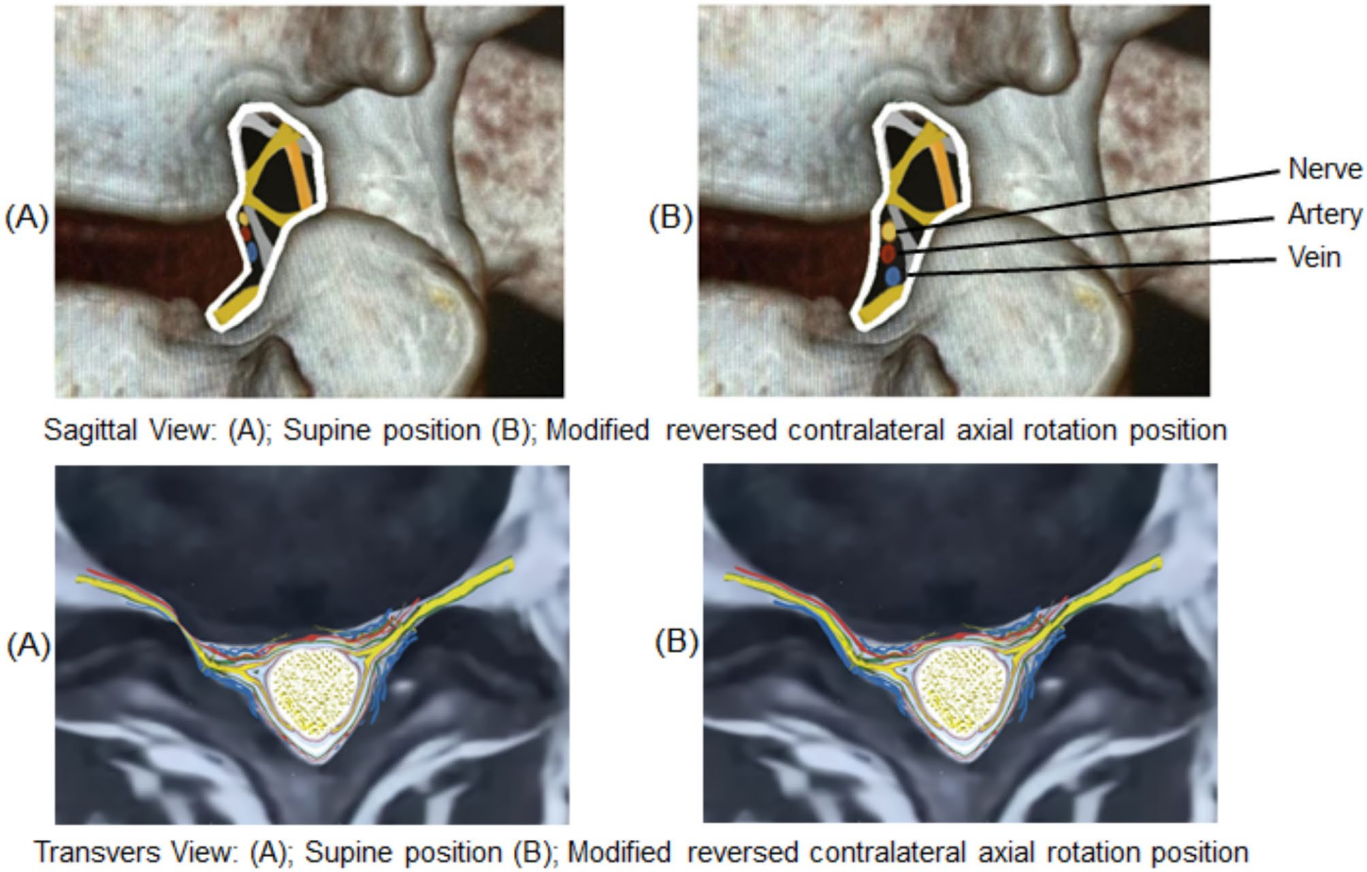
Schematic model of LIVF dimensional changes during the modified reversed contralateral axial rotation (MRCAR) and supine positions

Radiculopathy associated with unilateral lumbar disc prolapse is believed to be induced by mechanical nerve root compression, subsequent vascular disturbances [27, 28], and inflammatory mediators in extruded disc material [29, 30]. Obstructed blood flow, ischemia, disruption of normal homeostasis of the nerve root tissue, and/or inflammation of the nerve root/dorsal root ganglion (DRG)/spinal nerve complex are the most plausible pathophysiological mechanisms by which mechanical compromise causes radiculopathy [31, 32]. Under mechanical compression, nerve root tissue is exposed to degeneration, causing macrophages to release chemical mediators that worsen the radicular symptoms in the area of Wallerian degeneration [27]. Increasing the size of the LIVF could alleviate radicular symptoms and restore neurological function by relieving direct contact pressure on sensitized nerve tissue [30, 33] (Fig. 9).

**Fig. 10:**
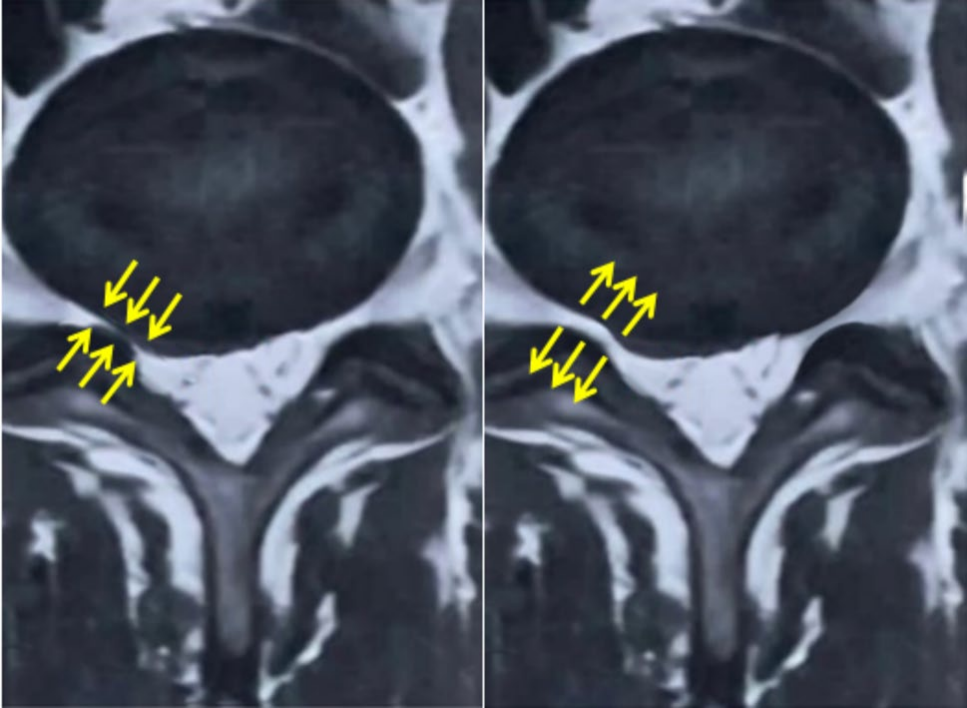
Lumbar MRI images, showing the LIVF at L5/S1. The right image depicts the modified reversed contralateral axial rotation (MRCAR) position, whereas the left depicts the supine position

The current study found that the MRCAR position significantly increased the range of SLR test compared with the supine position in all tested groups. This suggests that the MRCAR position may alleviate the nerve root compression and the resulting circulatory compromise, allowing for better nerve root mobility. This was evident in a case from group C, where an MRI examination of the lumbar spine showed nerve root decompression in the MRCAR position compared with the supine position (Fig. 10).

Ectopic impulse generation is proposed to be caused by endoneurial oedema in the DRG following mechanical compression of the herniated disc [31, 34]. Enlarging the opening of the LIVF by positioning could alleviate pressure on the DRG and/or spinal nerves, reducing ectopic impulse firing from the generating site, and thus alleviating radicular symptoms [31].

**Fig. 11:**
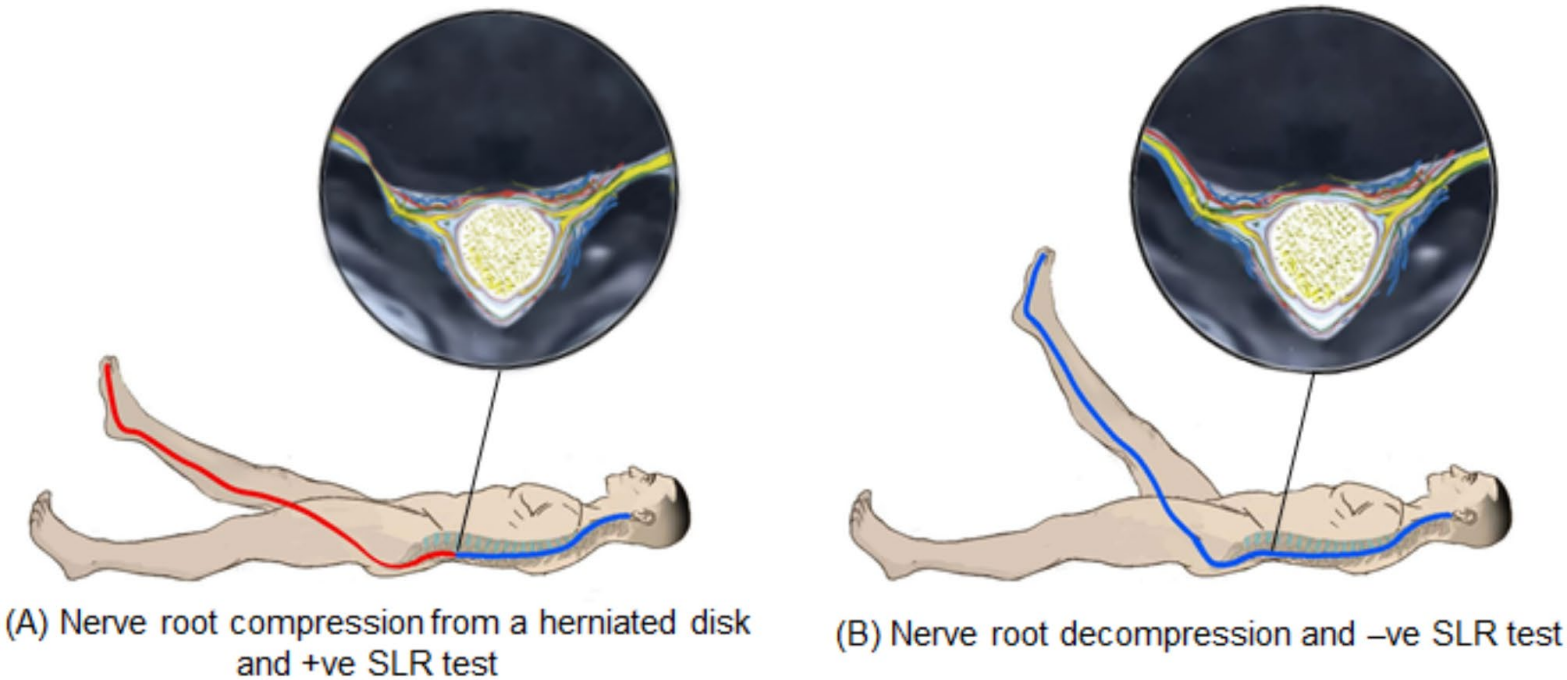
Schematic model of LIVF dimensional and SLR testing angle changes after releasing the nerve root compression

Nerve root inflammation/compression may reduce SLR range (< 70°) [31] via two mechanisms. First, it generates sufficient nociceptive discharges to stimulate the reflex muscle activity, particularly driven to the hamstring alpha-motor-neurons (muscle mechanism) [35]. Increased hamstring EMG activity has also been reported in response to SLR testing, supporting the hypothesis of protective muscle activity in patients with radiculopathy [36]. Second, it generates ectopic and nociceptive impulses to the inflamed/compressed nerve, as a result of increased sensitivity to stimuli due to tension/stretch (nerve mechanism) [37]. Positioning has additionally been shown to enhance the painfully limited SLR range, most likely by increasing the circumference of the LIVF, which decompresses neural tissue and reduces neural sensitivity to movement [31] (Fig. 11).

Furthermore, positioning may normalize nerve conduction values, sensation, reflexes, and muscle power by restoring normal conduction velocity of large-diameter myelinated afferent and efferent nerve fibers by increasing the LIVF diameter, which likely improves blood flow in spinal nerves and intraforaminal blood vessels, thereby reducing existing ischaemia, and in turn, clearing inflammatory exudate [31].

Previous imaging studies have demonstrated that even small increases in LIVF CSA can lead to decreased intraforaminal pressure, enhanced nerve conduction and better clinical outcomes for patients with lumbar foraminal stenosis [38]. An increase of 10–15% in LIVF CSA is linked to reduced nerve root compression to a degree that correlates with pain relief and improved function [39]. Moreover, an increase in SLR angle is often associated with reduced neural tension and enhanced nerve mobility, potentially resulting in symptom alleviation. An enhancement of 10°-15° in SLR is linked to functional recovery and pain reduction in patients with lumbar disc herniation [40, 41]. In our study, the observed increase in LIVF CSA and SLR angle exceeds past ranges, reinforcing the hypothesis that MRCAR positioning could clinically aid in improving functional capabilities and pain management.

The MRCAR position is in the opposite direction to movements and postures commonly used to initiate the centralization phenomenon in patients experiencing referred pain into the lower extremity. Typically, the centralization phenomenon is observed with lumbar extension with or without added ipsilateral side bending [42–45]. The centralization phenomenon has a known relationship to discogenic pain, providing a useful guide to the direction of mechanical loading that leads to lasting reduction of referred pain [46, 47]. In the event that extension and ipsilateral side bending causes peripheralization of symptoms rather than centralization of symptoms [48], it may be that the MRCAR position should be trialled as a therapeutic intervention instead. The MRCAR position may also serve as an alternative to the prone or side-lying positions that patients can assume during application of various physiotherapeutic modalities and can be included in a home exercise routine.

There were some limitations in the current study. At first, the study included only young male volunteers with unilateral L3/L4, L4/L5, and L5/S1 disc prolapse; hence results may vary for older individuals, females, and those with different lumbar spine issues. Second, it was ethically challenging to create a comparative group of asymptomatic individuals (control group) due to the high number of CT scans taken within a brief timeframe. Third, the MRCAR position was described as a possible non-surgical treatment option, but only showed a radiological change within 48 h. Therefore, future research needs to include long-term clinical data. Ultimately, even though the MRCAR position was standardized, there may still be potential inter-operator variability in attaining the proper MRCAR position.

## Conclusion

The modified reversed contralateral axial rotation (MRCAR) position of the trunk opens up the LIVF at L3/L4, L4/L5, and L5/S1 levels, improves SLR range of motion in the short-term (48 h), and has a real-time decompression effect on the impinged nerve roots. As a novel finding, it has potential clinical value as a manual positional nerve root decompression treatment technique for patients with unilateral lumbar disc prolapse and radiculopathy who do not respond to other conservative measures.

## Data Availability

No datasets were generated or analysed during the current study.
